# Cesarean childbirth: an institutional socio-clinical study of the professional practices and discourses

**DOI:** 10.1590/1518-8345.4756.3508

**Published:** 2021-11-19

**Authors:** Simone Santana da Silva, Cinira Magali Fortuna, Gilles Monceau

**Affiliations:** 1 Universidade do Estado da Bahia, Campus VII, Senhor do Bonfim, Bahia, BA, Brazil.; 2 Scholarship holder at the Programa de Apoio à Capacitação Docente e de Técnicos Administrativos da Universidade do Estado da Bahia, Brazil.; 3 Universidade de São Paulo, Escola de Enfermagem de Ribeirão Preto, PAHO/WHO Collaborating Centre for Nursing Research Development, Ribeirão Preto, SP, Brazil.; 4 Université de Cergy-Pontoise, Gennevilliers, France.

**Keywords:** Cesarean section, Parturition, Nursing, Health, Education, Social Sciences, Cesárea, Parto, Enfermería, Salud, Educatión, Ciencias Sociales, Cesárea, Parto, Enfermagem, Saúde, Educação, Ciências Sociais

## Abstract

**Objective:**

to analyze how the social, historical, economic and professional elements influence the institutionalization process of cesarean childbirth in Brazil and France.

**Method:**

a qualitative study grounded on the theoretical framework of the institutional socioclinic. The data were produced through focus groups and individual interviews, supported by semi-structured scripts, with health professionals, mothers and fathers and a research diary. The study had 83 participants. The analysis was based on the theoretical framework and thematic analysis. Cross analysis was developed between the different types of data, services and groups, as well as between countries.

**Results:**

the participants were characterized in terms of schooling, employment, level of training and workplace. The countries researched have their own peculiar health and educational structure and are marked by a social-historical-economic construction outlined by patriarchy. The New Public Management influences both contexts and makes health care precarious. The technical and organizational dimensions reinforce an intellectual and social division of work and knowledge in health.

**Conclusion:**

the institutionalization process of childbirth in the countries is marked by social, economic and professional aspects. Practices and speeches of the professionals in their work process influence the occurrence, or not, of cesarean sections.

## Abstract (es)

**Objetivo:**

analizar cómo influyen los elementos sociales, históricos, económicos y profesionales en el proceso de institucionalización del parto por cesárea en Brasil y Francia.

**Método:**

estudio cualitativo basado en el marco teórico de la socioclínica institucional. Los datos se obtuvieron a través de grupos focales y entrevistas individuales, basados en encuestas semiestructuradas con profesionales de la salud, madres y padres y en un diario de investigación. El estudio contó con 83 participantes. El análisis se basó en el marco teórico y el análisis temático. Se realizó un análisis cruzado entre diferentes tipos de datos, servicios, grupos y entre países.

**Resultados:**

los participantes fueron caracterizados por educación, empleo, nivel de formación y lugar de trabajo. Los países estudiados tienen una estructura sanitaria y educativa peculiar y se caracterizan por una construcción socio-histórico-económica basada en el patriarcado. La Nueva Gestión Pública influye en ambos contextos y precariza la asistencia sanitaria. Las dimensiones técnicas y organizativas refuerzan una división intelectual y social del trabajo y del conocimiento en salud.

**Conclusión:**

el proceso de institucionalización del parto en los países está marcado por aspectos sociales, económicos y profesionales. Las prácticas y los discursos de los profesionales en su proceso de trabajo influyen en la realización o no, de la cesárea.

## Abstract (pt)

**Objetivo:**

analisar como os elementos sociais, históricos, econômicos e profissionais influenciam o processo de institucionalização do nascimento por cesariana, no Brasil e na França.

**Método:**

estudo qualitativo fundamentado no referencial teórico da sócio-clínica institucional. Os dados foram produzidos por meio da realização de grupos focais e entrevista individual, apoiados em roteiros semiestruturados, com profissionais da saúde, mães e pais e diário de pesquisa. O estudo contou com 83 participantes. A análise foi feita com base no referencial teórico e análise temática. Desenvolveu-se análise cruzada entre os diferentes tipos de dados, serviços, grupos e entre países.

**Resultados:**

os participantes foram caracterizados em relação à escolaridade, emprego, nível de formação e lugar de trabalho. Os países pesquisados têm sua estrutura sanitária e educativa peculiar e são marcados por uma construção sócio-histórica-econômica delineada pelo patriarcado. A Nova Gestão Pública influencia ambos os contextos e torna os cuidados de saúde precarizados. As dimensões técnicas e organizacionais reforçam uma divisão intelectual e social do trabalho e do saber em saúde.

**Conclusão:**

o processo de institucionalização do nascimento nos países é marcado por aspectos sociais, econômicos e profissionais. Práticas e discursos dos profissionais em seu processo de trabalho influenciam a ocorrência, ou não, da cesariana.

## Introduction

For a long time, delivery was an intimate experience, which took place at home, vaginally and accompanied by midwives or people with traditional knowledge. In the period before the eighteenth century, childbirth was not thought of in any other way than the established one^(1)^. Different dimensions, such as social, historical, cultural, political and economic, associated with the advancement of the technologies, demarcate strong influences in the context of maternal and child care, while hospital-centered interventionist practices began to expand. In this process, medical knowledge takes control of deliveries and this is reflected in the increased occurrence of cesarean interventions^(1-3)^. It is recognized that, when adopted under ideal and necessary conditions from a biological point of view, the cesarean intervention reflects positively on the maternal and neonatal morbidity and mortality rates^(1)^.

This transformation that has taken place is a dialectical process in which social structures are under the action of forces that provoke constant changes, the instituting forces. The action of instituted and instituting forces causes social dynamics and promotes constant changes, called institutionalization^(4)^.

Currently, in the world context, the debate about childbirth is marked by the effort to reduce the number of cesarean interventions and, at the same time, by the defense of the provision of qualified care to women^(5-7)^. In Brazil, in the last 40 years, there has been a marked increase in the number of surgical deliveries associated with high rates of preterm childbirth and prematurity with iatrogenicity^(3,8)^.

In this production, supported by the institutional socio-clinical framework^(9)^, a result of the French Institutional Analysis (IA)^(10)^, maternal and child health is understood as an institution that operates based on the precepts validated in policies and legislation. It even integrates the professional protocol practices, also instituted. IA clarifies how the institution’s transformation process, institutionalization, is cross permeated by active contradictions, which bring about contributions from the participation of subjects^(4,10-11)^. The notion of object group and subject group^(10)^ contributes to the analytical process of the institutional interventions outlined in the framework. The object group is the one that does not question much, identifies itself with the aspects instituted in the institution and is a resource that contributes to its existence. Inversely, the subject group acts in the search for strategies that deviate from the instituted^(10)^.

Brazil is among the countries in the world that performs a high number of unnecessary cesarean interventions^(1,3,5,7,8)^. France has low rates of cesarean operations; however, social and professional groups are concerned about the increase in interventions around the world, the so-called “active cesarean sections” and the growing trend of the so-called “cesarean sections on demand by women”^(12-13)^. The reflection about performing unnecessary cesarean sections includes the influence of the technocratic models in the construction of the idea of safety and protection in the social imaginary related to delivery^(1,5,7,12)^.

Given above, the study aims at analyzing how social, historical, economic and professional elements influence the institutionalization process of cesarean childbirth in Brazil and France. There is no intention to carry out a comparative study between the countries since, eventually, each of them has its peculiarities in its constitution, history and context. The intersection of the different realities studied can produce analyses explaining barely visible aspects and recognizing specificities and differences. This study is the result of a multicenter research study, which deepens issues related to childbirth and parenting in Brazil, Canada and France^(14)^.

## Method

### Study design

This text follows the guidelines of the Consolidated Criteria for Reporting Qualitative Research (COREQ), which seeks to ensure the quality of the description of the research stages. It is a qualitative study, guided by the theoretical-methodological framework of IA, in line with the French Institutional socioclinic^(4,9-11)^. Among the characteristics of institutional socioclinic, there are the following: Working on the ordering and demands; participation of subjects in the approach in variable ways; working on the elements that reveals hidden issues of the institution; analysis of the changes produced as the work progresses; implementation of restitution modalities that return provisional results of the work to the research participants; working on the primary and secondary implications with the people involved in the process; prediction of knowledge production; and attention to the institutional contexts and interferences linked to the researchers and participants^(15-17)^.

Data production was initiated with the research diary written by the first author throughout the research. As already mentioned, the study is articulated with a multicenter study^(14)^ that organized focus groups to deepen the dialog on childbirth and parenthood. Based on the arguments of these groups, for this study, the information that dealt with the experience with childbirth was selected. Finally, another data production strategy occurred through individual interviews. The first author participated in all the data production stages in both countries.

### Research locus and development period

The research was carried out in the city of Ribeirão Preto, SP, Brazil, and in the Hauts-de-Seine and Val d’Oise regions, in France, between 2016 and 2019.

### Participants, selection criteria and procedures for data collection

The participants were professionals working in maternal and child care services, members of associations concerned with the health of mothers and infants and mothers and fathers.

While selecting the participants, priority was given to the desire and availability to participate, being over 18 years of age and formalizing participation by signing the Free and Informed Consent Form (FICF). The mothers and fathers interested in participating should have gone through the experience of pregnancy and childbirth, regardless of the outcome. In turn, the professionals should work in the maternal and child public health care sector.

To assemble the focus groups, the following criteria were adopted: two groups constituted by parents coming from different social, economic and educational contexts classified as: “Social 1” and “Social 2”. In Brazil, they were represented by a public daycare center (Social 1) and a language school (Social 2). In France, the “Social 1” group consisted of mothers and fathers who used a service that served families, called “*Maison des familles*” and the “Social 2” group was constituted by an association of parents.

Organization of the group of professionals was based on the following criteria: two groups of public servants with different profiles, classified as “hospital” and “other institution”. The “hospital” group, coming from the Brazilian context, was a hospital care service for women, and the focus group called “another institution” consisted of a reference hospital for maternal health. In France, the groups were made up of professionals from a maternity ward and a mother and child protection service, referred to as “*Protection Maternelle Infantile*” (PMI). Three meetings were held with each group.

The total number of participants was 83, including professionals, mothers and fathers, as well as members of associations concerned with the health of mothers and infants. The distribution was as follows: a) focus groups by country: 4 groups, consisting of 8 individuals each (32 people in Brazil and 32 people in France); b) individual interviews: 09 Brazilian mothers (2 not belonging to the focus group), 10 Brazilian professionals (4 were not part of the focus group); 07 French mothers (4 did not participate in the focus group), 02 French couples (none participated in the focus group), and 09 French professionals (5 did not participate in the focus group). In Brazil and France, both focus groups lasted approximately 1,400 minutes. The individual interviews lasted a mean of 900 minutes. Data validation was performed by rereading the transcripts and discussing the material with the research team, guided by the study objectives and the theoretical-methodological framework.

In Brazil, professionals working in maternal and child care services participated, including those who were part of the municipality’s Maternal and Child Mortality Committee (*Comitê de Mortalidade Materno-Infantil*, CMMI). In the French reality, professionals working in the regions of Hauts-de-Seine and Val d’Oise participated, as well as members of associations concerned with the health of mothers and infants.

In relation to the individual interviews, mothers and fathers were invited, specifically mothers or couples who took part in the focus groups. Agreement to participate in this new moment of the research was not unanimous; for this reason, women or couples were asked to indicate another mother or couple from their social group and who underwent the experience of giving birth, regardless the delivery outcome. The individual interviews with the professionals were initiated by the members of the focus groups and CMMI professionals. It is important to clarify that the first author participated in the CMMI case discussion meetings and, when appropriate, invited the members to take part in the research. As the members of that committee worked in the municipality’s mother and child care network, they were part of the professional categories.

A semi-structured script was developed to support conduction of the groups and for the interviews.

### Data treatment and analysis

The analysis was supported in the approach of Paillé and Mucchielli’s thematic analysis^(18)^, which has the argumentation stage of the theme concretized in a qualitative, interpretive and hypothetical exercise.

**Figure 1:**
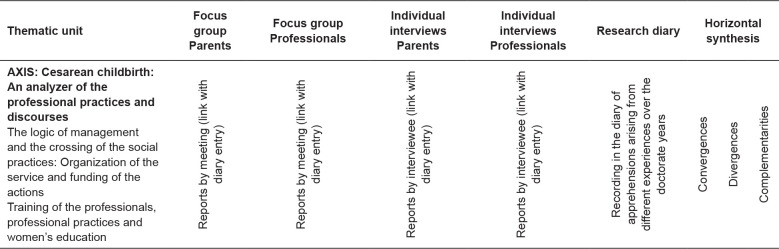
Analytical chart of the empirical data resulting from the focus groups, interviews and research diary. Brazil and France, 2016-2019.

Based on the data transcriptions, a hybrid approach to the analysis was adopted, in which continuous thematization and sequential thematization were included. Continued thematization consists in assigning, regrouping and adjusting the themes progressively until the end of the apprehensions. In turn, sequential thematization offers support for carrying out analyses with approximations and distances between groups. In summary, the moments that supported the analytical process were as follows: 1- transcription^*^ (listening to and transcribing the groups); 2- transposition (constituting the units of meaning and the approximations between them and the theoretical frameworks); 3- reconstitution (rebuilding a text with the final analyses). Figure 1 illustrates the organization process of the analytical stage of this research.

The research diary notes were intertwined with the syntheses from the groups and the individual interviews. Horizontal summaries were written from the records resulting from each data production category. Convergences, divergences and complementarities were demarcated.

Also in the analytical process, there was participatory restitution, a moment provided for in the framework employed, which consists of the presentation of provisional results to the participants. This stage took place through: 1- sharing the parents’ and professionals’ experiences, in different groups and countries, in relation to the perinatal period; 2- conversation about the elements that support the professional practices and social conceptions in relation to the theme. This dialog provoked reflections and constituted an educational component of the research. The moments of participatory restitution can enable the study participants to continue and mutually analyze, in addition to prompting them to reflect on their own practices, conceptions and experiences^(11,15,19)^.

### Ethical aspects

Data production in Brazil was initiated after authorization from the co-participating institutions and approval by the Research Ethics Committee (opinion No. 2.623.731).

Identification of the participants in the focus group meetings was SOCIAL 1 or SOCIAL 2 for the group of parents belonging to the researched devices, and HOSPITAL or OTHER for the groups trained in the women’s assistance services. An Arabic number was added to identify the meeting and also BRA or FRA to recognize the country to which it refers to (for example: SOCIAL 1 - 0/BRA). For each interviewee, the following identifications were adopted: MOTHER/MÈRE, FATHER/PÈRE and PRO for mothers, fathers and professionals, respectively, followed by an Arabic numeral and BRA (Brazil) or FRA (France). The identification MÈRE/PÈRE, mother and father in French, has been retained in the empirical material to assist the reader in contextualizing the participants’ speech in relation to their nationality. In addition, it is clarified that, due to non-full transcription of the individual interviews carried out in France, the following coding was adopted: Individual interview report, followed by FRA and MÈRE or PRO and an Arabic number (for example: Individual interview report/FRA/PRO/05). For the excerpts from the research diary, the following coding was adopted: search diary, location, country, date.

Although in France the same ethical criteria are not followed, in Brazil authorization of the researched care device and of the participants was not waived. Confidentiality of the information shared was ensured and adoption of signature of the FICF was maintained. The authorizations were obtained from the local authorities and the people who took part in the research. Regarding the groups of parents assembled, in France, as they do not meet in health services, organizational authorization was not requested, only individual consent.

## Results

It is verified that the socioeconomic profile of the members of the focus groups of mothers and fathers assembled in the countries is quite heterogeneous. In relation to the focus groups conducted, in France, the mothers’ and fathers’ age varied between 35 and 46 years old. Among the women belonging to the *Maison de Familles* Service group, the largest number were immigrants of North African origin, unemployed and without university schooling or with some higher education not recognized in France. On the other hand, the *Maison de Nassance* Association group consisted of psychologists, social workers, nurses and an engineer. They had higher income and schooling characteristics, with a salary level between 2,000 and 3,000 euros *per* month^*^ and BAC^**^ +3 and BAC +5 educational levels. The number of children varied from one to four.

No foreigners participated in the research in Brazil. The daycare center group was consisted mostly of women with a job contract and incomplete or ongoing university studies. In the group of participants from the Language School there were a lawyer, professors, a nursing technician, an agronomist and an administrator, with high schooling and economic levels and earning more than R$3,000 *per* month. The women’s age varied between 30 and 72 years old and most of them had already experienced a cesarean section. Also in relation to the group, in addition to the Unified Health System (*Sistema* Único *de Saúde*, SUS - The Brazilian public health system), most of them had a private health plan.

The focus groups constituted by French professionals consisted of *sage-femmes* ^***^, an obstetrician and gynecologist, a childcare specialist (*puericultrice*), a nurse, a childcare assistant, a Nursing student and a PMI secretary. Regarding length of experience, it varied from 2 to 31 years and the majority did not have more than one employment contract. In the Brazilian context, the focus groups consisted of psychologists, speech therapists, an occupational therapist, nurses, social workers, a physiotherapist, two medical residents, and a nursing assistant and technician.

In relation to the individual interviews conducted with mothers or couples in the French context, two women are of Moroccan origin, one being a housewife and the other one, unemployed. The professional qualification level of these women was lower than that of the other participants (BAC 0 and BAC +3). The other interviewees had a schooling level equal to or higher than BAC +5. Regarding the type of delivery, 06 French women underwent cesarean sections.

The profile of the mothers interviewed individually in Brazil revealed that the number of children varied between 1 and 5. In addition to the SUS, most of them had a private health plan. Only two women used the SUS exclusively, both black-skinned and single. In relation to the monthly income, three women, including the two mentioned above, had a salary below R$1,000 or between R$1,000 and R$2,000^****^. Most of them underwent cesarean surgeries and only 03 of the women interviewed individually experienced vaginal deliveries.

For the category of professionals interviewed individually, in Brazil, their age varied between 32 and 69 years old, most were female and their length of professional experience ranged from 3 to 44 years. Three individuals had more than one employment contract and the others had an effective contract in the public service. The individuals interviewed in France were aged between 29 and 57 years old, most were female and their length of professional experience varied between 2 and 34 years. One person had more than one employment contract (public and private) and the others worked in the French public service.

The research results revealed that “truth speeches”, as power devices, are unveiled in the professionals’ practices and speeches throughout their performance. This influences the institutionalization of childbirth by cesarean section and makes visible the robustness with which power acts on the relationships, organizations and institutions. Another element that interferes in the structuring of discourses and practices is the financing system for health actions and the logic in which they are structured in the actions provided. The following lines confirmed such arguments: *In the large university services there are many births and this is proportional to hypermedicalization, because there’s no time... people keep coming. This makes a way of working mandatory... “I medicate more”... which reflects on the outcome by cesarean, because in certain cases, childbirth requires waiting a long time... 24 hours, 48 hours and that’s too long (in the financing logic). And in the modern hospital services, the money that comes in depends on the activity, on the profitability of the service, and this doesn’t allow “holding” people 48 hours in the service, doing nothing, otherwise money is lost.* (Report of individual interview/PRO/FRA/01).

In Brazil, arguments given by the participants in relation to the professionals’ practices and discourses and the reflections produced reveal their specificities as in the following speech: *So, vaginal childbirth, normal is the delivery that should be habitual (...). The problem is that over a period of time, a significant number of women died during delivery. That’s when people started to develop the cesarean section, which was difficult (...) it was almost signing the woman’s death certificate because of the surgery. But the thing was gradually organized in such a way that today a cesarean is a very simple, easy and low-risk methodology. The problem is that no one’s abdomen is meant to be opened, it was developed for emergency care, where the child or mother is between life and death. Oh, so it is justified (application of the technique). And when you develop, it’s wonderful, easy, comfortable, I don’t have labor, I don’t have labor contractions, I don’t have the difficulties of delivery and it’s still easy to do? Fast, good for the doctor [...]* (Individual interview PRO/BRA/01).

In France, where most deliveries occur vaginally, the most evident tensions were in the order of the medicalization of actions, that is, the excessive burden of interventions on the female body in the parturition process. *I said: “let’s try to make him breastfeed more to see if it helps the placenta...” then came another sage-femme ... one tried, couldn’t, another came, then tried to put it on and he sucked a little... I don’t know, two minutes, you know? And it came out of the breast and couldn’t latch any more... and the placenta wouldn’t come out! So... there was a time when the sage-femme started pulling my placenta by the umbilical cord... and it broke (ruptured the placenta)* (MÈRE/FRA/03).

Another result demarcated in the research was in the field of the social inequalities and their interference in the assistance to mothers and infants. Brazil, a country vigorously affected by inequalities, revealed peculiar aspects in its unfolding and expansion in contexts that aggregate different vulnerabilities. Consequently, these inequalities mark the practices of delivery care. This was revealed, for example, in the lives of women who, on the one hand, use the SUS for prenatal, delivery and puerperium care, but who were unable to actively participate in the decisions related to the actions taken by the professionals who assisted them. Also in the Brazilian context, but in another position, were those women users of private health plans, often captured by the financial and highly interventionist logic, which supported in their speeches the idea of safety in the care offered by the professionals and in the outcome of surgical deliveries. There are also the situations of women who want vaginal delivery and, in order to experience it, in some cases need to overcome the constraints in the professionals’ discourse, operating logic of the contracted operator and even assume extra financial costs of the procedure, even if they are holders of private medical insurance plans. The following testimonies exemplify such situations: *But I really wanted my delivery to be normal. In the fifth month I found out that she [the doctor] didn’t do normal deliveries, but I had deified her in a way (...) that the day I found out she didn’t do normal deliveries... okay, I’m going to do a cesarean. Like not... (...) for me she was a myth, she was going to save my daughter, you know?* (MOTHER/BRA/06). The following speech also highlights elements discussed: *[Mother 06] [...] When I spoke to the doctor: “I want to have a normal delivery”, he said: “Do you want to feel pain for at least 24 hours?” (...). At no time did he encourage me. From the moment I said I wanted a normal delivery, he didn’t. Why? Because nowadays they don’t get paid for that. Then with my second child, I have no insurance, right? I went to the SUS [...] I had a humanized delivery at [says the name of the service], my husband sitting behind me on a stool, you know? My dream came true without me spending any real money. So, many criticize the SUS, but I had a humanized delivery that I would pay R$ 6,000 (if it was in the private service)* (Excerpt from the parents group meeting 01/DAYCARE CENTER/BRA).

In the French context, arguments related to the professional practices and discourses aimed at delivery illustrate acknowledgment of the tensions of the debate by those who work in the care of women and children: Stresses that even conventional delivery in France is quite medicalized, there are many interventions. So... she already imagines herself lying down, legs apart, with several different people, touching and maybe having an episiotomy practiced. And maybe it’s great anguish. So it’s hard for her to find a professional who is listening (Report of individual interview/PRO/FRA/02). In the Brazilian context, although less frequently, recognition of the weaknesses of their practices and discourses was also present as stated in the following speech: *In the public service, the woman doesn’t have many options, the attempted delivery route is normal delivery. If she wants a C-section, she doesn’t have a choice, let’s try normal. If it isn’t effective, you go for a cesarean. So much so that we have a difference in the rate of cesarean sections between public and private. In the private sector, the woman already has a choice. But these choices are made without guidance* (Individual interview PRO/BRA/02).

## Discussion

In view of what is pointed out in the results, it is possible to highlight that childbirth by cesarean section is an analyzer of the professional practices and discourses. The contradictions of an institution are revealed by the analyzers, which can be an object, an event, an individual or a piece of information, among others. They find a way to give visibility to the camouflaged aspects of social life and often reveal themselves in more ambiguous and less vivid ways^(19)^. In this production, it is verified that childbirth is influenced by different places and contexts. It is marked, for example, with the logic of the management model of the health services in the countries and the concept of funding, which interpenetrate between the professional practices. Such interferences reflect in the professional actions, in the speeches and in educating women when preparing them for delivery.

The SUS production as a policy is permeated by tensions among managers, workers and users. In this order, the last two actors are regularly understood as those doomed to obedience. The starting point is the understanding that, in practical terms, everyone assumes their leading role in management, contributes to the formulation of strategies and all operate disputes, even if in the field of invisibility or without explanation of the different projects^(20)^. It cannot be denied that the policies produce regularities, standardization and captures that generate a uniform pattern that can immobilize. The legal and regulatory frameworks that outline maternal and child health indicate the intention to achieve a humanization of delivery care that goes beyond the established care model, that is, marked by the empowerment of human life through Medicine and interventions on the body. In this case, the intention is to reduce the weaknesses resulting from the hospitalization of delivery. The implementation of the Brazilian Policy for the Humanization of Delivery, for example, is weakened by the shocks linked to the care model that is put into practice. These shocks can be of a professional nature, especially in Medicine in relation to other categories, such as obstetric nurses, midwives, and even the inclusion of categories such as doulas; organizational, which are connected to the protocols instituted in the services and to the “induced demands” created by the services themselves in a logic of productivity; and policies that are associated with the interventions and interests of the public power that tends to be focused on the results produced.

In each context under study, even with its peculiarities, social disparities as well as health and access inequalities are issues related to the care provided. This occurs because women in precarious situations may not be accompanied during pregnancy or may be following fragile prenatal care. As a result, they tend to incorporate the prevention strategies in a limited manner^(2,21)^. In both countries, deliveries take place mostly in hospital spaces. The power exercised by the professionals who monitor the women in the prenatal, delivery or postnatal periods in the consultations, complementary exams or in the guidelines, in general, ensures the permanence of a logic of divisions and ultra-specialization of the care practices, the “fordist” assistance actions. Such reality reflects in the institutionalization process of childbirth, including preservation of violent actions, such as those in the gender or organizational field and marked by delivery acceleration or withdrawal of women’s autonomy. Given the above and other apprehensions demarcated by the research, it is understood that the hospital actions offered, whether at the reception of women, in their comfort, or even in the degree of appreciation of the mother and infant’ unique process in the assistance services, are strongly influenced by quality seals, such as the Women and Baby Friendly Hospital Initiative (*Iniciativa Hospital Amigo da Criança*, IHAC)^*^. The aforementioned seals, in many services, the decisive factor for changes in the care process and in the degree of interventions performed^(13)^. This means that the service management method leads to the quality of care offered to people^(22)^. Thus, existence of a risk in this process, which can be, for example, signaled by the logic of the New Public Management, cannot be denied. It values the private initiative model and supports an idea of efficiency, although it negatively affects daily work and the relationships^(23-24)^.

The research results contribute to the advancement of scientific knowledge, as they reveal the action of instituted and instituting forces, which influence the professional practices in naturalized actions and in the consequences related to the advancement of the number of births by cesarean section. They reflect on the woman’s and infant’s vulnerability in the face of the surgical process itself, on the difficulty of offering humanized care, and on the submission of the pregnant woman and infant to strict routines. The support of institutional socioclinic allowed understanding macrosocial and microsocial aspects in the theoretical and social genesis of cesarean births, brought up reality and pointed out trends in births in each country.

Among the study limitations, there is the lack of depth in aspects related to the variables related to race, skin color and ethnicity of the women and their influence on the institutionalization of births that occurred by cesarean in both countries. In addition to that, the study did not address aspects related to the training of the professionals involved, including the reproduction of body control practices by Nursing in the care of mothers and infants.

## Conclusion

Social, historical, economic and professional aspects demarcate, even if differently, the debate related to cesarean childbirth in the Brazilian and French contexts. In these countries, the occurrence of cesarean sections reveals contradictions in the professional practices and discourses and, as a consequence, tends to contribute to the women’s degree of consent in relation to the control of their bodies.

Among the social aspects involved, it is evident that, whether in Brazil or in France, social disparities, as well as health and access inequalities, are issues related to assistance to women and infants. With regard to the economic aspects, the private initiative model is installed in the public services and influences the understanding of daily work, and the relationships and outcomes of the deliveries, which opens up space to amplify the disputes that exist in this care model. Those of a professional nature, considering the professional modalities of each country, mark how the individual body is transformed into a workforce, based on structured knowledge, which, in some situations, devalues the intimate aspects of the pregnant woman. In the Brazilian case, this dispute is often fought between Medicine and Nursing. In France, in turn, between physicians and *sage-femmes* in the private services.

A dimension that emerges from the problem presented with this research on unnecessary cesarean interventions is the analysis that the strong medicalization in childbirth and the growth in cesarean rates can contribute to women questioning the current obstetric model in the countries and seeking information in search for their autonomy through an informed choice. They can therefore incorporate resistance strategies to the instituted. The French professionals show more open recognition of problems related to their practices, as well as the resistance implemented in front of them.

Women can decide to run the possible risks, resist and create strategies to claim their desires. In relation to these desires, it is necessary to clarify that the starting point is the understanding that they are also built based on the professionals’ practices and discourses, specific experiences and culture, among others. Regarding the debate on autonomy and delivery, the study points out that French women seem to be closer to the appropriation of their bodies and desires.

## Footnotes

* Not all the individual interviews conducted in France were transcribed. For those not transcribed, synthesized texts were used.

* Current minimum wage = €1,521.22, France, 2019.

** BAC: *Baccalaureate*, Bachelor’s degree in English. It is a degree obtained through an exam performed after the *lycée*, secondary education, for access to higher education. After that, the next 3 years are the *licence* classified as BAC +1, BAC +2 and BAC +3. The last two years of university studies are the *master* 1 or *master* 2, which will guarantee BAC +4 or BAC +5, respectively. The students holding the M2 diploma can apply for the PhD that guarantees the BAC +8.

*** We kept the French terms in these professions because there is no similarity of training and professional practice in Brazil. *Sage-femme* is a health professional graduated in a specific five-year course at the university with first-year training together with the medical course. A *puericultrice* is a nurse or *sage-femme* who specializes in early childhood care. After initial training she must pass a competitive examination in a specific school of childcare. Names of regions and services were also kept in the French language.

**** Current minimum wage = R$998,00, Brazil, 2019.

* Seal of quality granted by the Ministry of Health to hospitals that comply with the 10 steps to successful breastfeeding, instituted by UNICEF (United Nations Children›s Fund) and by the World Health Organization.
